# Morin Inhibits Dox-Induced Vascular Inflammation By Regulating PTEN/AKT/NF-κB Pathway

**DOI:** 10.1007/s10753-022-01701-5

**Published:** 2022-06-15

**Authors:** Jing Yu, Hai-Liang Qi, Hong Zhang, Zi-Yu Zhao, Jing-Zhao, Zi-Yuan Nie

**Affiliations:** 1 Department of Hematology, The Second Hospital of Hebei Medical University, 215 Heping W Rd, Shijiazhuang, 050004 China; 2 Second Department of Respiratory and Critical Care Medicine, The Second Hospital of Hebei Medical University, Shijiazhuang, 050004 China; 3 Department of Thoracic Surgery, Hebei Provincial Chest Hospital, Shijiazhuang, 050047 China; 4 Department of Urology, The Second Hospital of Hebei Medical University, Shijiazhuang, 050004 China

**Keywords:** Doxorubicin, Morin, Vascular smooth muscle cells, PTEN, Vascular inflammation

## Abstract

The side effects of doxorubicin (Dox) may influence the long-term survival of patients with malignancies. Therefore, it is necessary to clarify the mechanisms generating these side effects induced by Dox and identify effective therapeutic strategies. Here, we found that interleukin-6 (IL-6), interleukin-1β (IL-1β), and tumor necrosis factor-alpha (TNF-α) levels were significantly increased in vascular tissues of Dox-treated mice and Dox-treated vascular smooth muscle cells (VSMCs). Furthermore, we revealed that Dox downregulated the phosphatase and tension homology deleted on chromosome 10 (PTEN) level while upregulated p-AKT and p65 level in VSMCs in vitro. Overexpression of PTEN in VSMCs partly reversed Dox-induced inflammation. Importantly, we demonstrated that Morin could inhibit Dox-induced inflammation by facilitating an increase of PTEN, thus inhibiting the activation of protein kinase B (AKT)/nuclear factor kappa B (NF-κB)/pathway. Additionally, we showed that Morin could reduce the miR-188-5p level, which was increased in Dox-treated VSMCs. Inhibition of miR-188-5p suppressed Dox-induced vascular inflammation in vitro. In conclusion, Morin reduced the Dox-induced vascular inflammatory by moderating the miR-188-5p/PTEN/AKT/NF-κB pathway, indicating that Morin might be a therapeutic agent for overcoming the Dox-induced vascular inflammation.

## INTRODUCTION

Worldwide, cancer by far is the fatal disease with the second highest mortality rate which leads to 606,520 deaths in 2020 in the USA [1]. Despite the development of novel diagnostic technologies, the emergence of targeted drugs, and the recent advancements in radiotherapy technology, chemotherapy remains the cornerstone of cancer treatment [2–5]. Pleasingly, in the last decade, approximately 50% patients with malignancies have been clinically cured and have a long-term survival due to chemotherapy [1]. However, part of survivors who have exposed to the chemotherapeutics may suffer from the long-term side effects, of chemotherapeutics which can adversely affect their quality of life [6–8]. Therefore, it is necessary to clarify the mechanisms generating these side effects and identify effective therapeutic strategies.

Doxorubicin (Dox) is an anthracycline antibiotic that has been used widespread in treatment of multifarious malignancies, including solid tumors and hematological neoplasms [9]. The most widely and popular research studies have focused on the Dox-induced cardiotoxicity [10, 11]. Nevertheless, the side effects of Dox are not merely restricted to cardiotoxicity, but also include hepatotoxic, nephrotoxicity, and vascular injury [12, 13]. Vascular inflammation ultimately leads to the development of cardiovascular disorders by dysregulation of proliferation and apoptosis of VSMCs, vascular remodeling, and fibrosis. More importantly, vascular inflammation is also the pathophysiology basis of organ dysfunction [14–17]. Therefore, elucidating the molecular mechanism of vascular inflammation induced by Dox and finding the therapeutic or prevention strategies are urgent requirements.

Morin (3,5,7,2',4'-pentahydroxyflavone) is a flavonoid which is separated from fruits and vegetables of the Moraceae family [18]. Extensive studies have confirmed that Morin has multiple pharmacological effects, such as anti-atherosclerosis, antidiabetic, anti-oxidative, anti-inflammatory, and anti-tumor activity by regulating the PTEN/AKT pathway [19–21]. Consistent with several other studies, our previous research has demonstrated that Morin has an anti-leukemia effect on leukemia cells by regulating miR-188-5p/PTEN/AKT pathway [22]. Therefore, we hypothesized that whether Morin could decrease the Dox-induced vascular inflammation by moderating the activation of PTEN/AKT pathway.

In the present study, we revealed that Dox could induce the vascular inflammation in vivo and in vitro by regulating miR-188-5p/PTEN/AKT/NF-κB pathway. Crucially, we revealed that Morin inhibited inflammation which induced by Dox by suppressing miR-188-5p and subsequently upregulating PTEN expression.

## MATERIAL AND METHODS

### Animal Model

All animal studies were approved by the local Animal Care and Use Committee of Hebei Medical University. Six- to eight-week-old male C57BL/6 mice were housed in a climatically controlled environment, and all efforts were made to minimize suffering. In the Dox-induced vascular inflammation models, male C57BL/6 mice were randomly divided into two groups, namely the control group and the Dox group, and each group contained eight mice. Mice were intraperitoneal injected with 6 mg/kg DOX twice a week for 2 weeks while the control group was treated with an equivalent amount saline. In the Morin-reduced Dox-induced vascular inflammation mouse model, male C57BL/6 mice were randomly divided into three groups, namely the control group, Dox group, and Morin combination with Dox group, and each group contained eight mice. In the Dox group or Morin combination with Dox group, mice were intraperitoneally injected with 6 mg/kg DOX twice a week for 2 weeks while the control group was treated with an equivalent amount saline. In Morin combination with Dox group, the mice were intraperitoneal injected with 25 mg/kg Morin five times per week for 2 weeks. Fourteen days later, all animals were anesthetized, and the abdominal aorta was taken. The vascular tissues were fixed in paraformaldehyde or stored at −80 °C for the future experiments.

### Immunohistochemistry

Immunohistochemistry was carried out as previously described [23]. The antibodies were used as follows: anti-IL-1β (1:100, Proteintech, 16,806–1-AP), anti-IL-6 (1:100, Proteintech, 21,865–1-AP), and anti-TNF-α (1:100, Proteintech, 6029–1-Ig). Images were acquired using a Leica microscope (Leica DM6000B).

### Immunofluorescence

The fixed smears of vascular tissues or VSMCs were permeabilized by incubation with 0.1% Triton X-100 in PBS for 30 min. After blocking by incubation with 10% normal goat serum (710,027, KPL, USA) for 1 h, the smears were incubated for 2 h at 37℃ with the following antibodies: anti-IL-1β (1:100, Proteintech, 16,806–1-AP), anti-IL-6 (1:100, Proteintech, 21,865–1-AP), and anti-TNF-α (1:100, Proteintech, 6029–1-Ig). The smears were washed with 0.1% Triton X-100 in PBS for three times and stained with secondary antibodies which were fluorescein-labeled (1:50, KPL, 021,516) and 4′,6-diamidino-2-phenylindole (DAPI). Images were captured by confocal microscopy (DM6000 CFS, Leica Microsystems).

### Enzyme-Linked Immunosorbent Assay (ELISA)

ELISA was performed in accordance with manufacturer’s instructions. Using a commercial ELISA kit (Proteintech, **KE10002, KE10003, KE10007**) to test the concentrations of IL-1β, IL-6, and TNF-α which obtained from mouse serum. The absorbance at 450 nm was detected with a Multiskan Ascent (SPECTRAFluor Plus, Tecan).

### Cell Culture and Transfection

Mice VSMCs were cultured in low-glucose Dulbecco’s modified Eagle’s medium (DMEM, Gibco Life Technologies, Rockville, MD) and 10% fetal bovine serum (GEMINI, USA) in the incubator at 37 °C with 5% CO_2_.

Cells were transfected with Lipofectamine 2000 (Invitrogen) according to the manufacturer’s protocols. The miR-188-5p inhibitor, si-PTEN, and PTEN overexpression plasmid were selected as previously [22].

### Isolation of RNA and Reverse Transcription-Quantitative Polymerase Chain Reaction (RT-qPCR)

Total RNA was extracted from the tissues or cultured cells by using QIAzol Lysis Reagent (79,306) according to the manufacturer’s protocol. Then, 2 µg RNA was used to reverse transcription reaction by using M-MLV First-Strand Kit (Life Technologies) for mRNA and the miScripIIRT kit (QIAGEN GmbH) for microRNA. Platinum SYBR Green qPCR SuperMix UDG Kit (Invitrogen) and miScript SYBR Green PCR kit were used qRT-PCR for mRNA and microRNA respectively according to the manufacturer’s instructions. As an internal control, β-actin and U6 were used for the internal control of mRNA and microRNA, respectively. Relative transcripts were calculated using the 2^−ΔΔCt^ method.

### Western Blot

Proteins from vascular tissue and cultured VSMCs were lysed by protein lysis buffer. Proteins were run on 8–10% SDS-PAGE to separation and then electro-transferred to PVDF membranes (Millipore). The membranes were blocked with 5% milk in TTBS for 2 h at room temperature and incubated overnight at 4 °C using the primary following antibodies: anti-IL-1β (1:1000, Proteintech, 16,806–1-AP), anti-IL-6 (1:1000, Proteintech, 21,865–1-AP), anti-TNF-α (1:1000, Proteintech, 6029–1-Ig), anti-PTEN (1:1000, Abcam, ab32199), anti-pan-AKT (1:1000, Abcam, ab8805), anti-pan-AKT (phospho T308; 1:1000, Abcam, ab38449), and 1:1000 anti-β-actin (1:2000, Santa, sc-47778). In the next day, after washing in the TTBS for three times, membranes were incubated with a 1:5000 dilution of anti-rabbit or anti-mouse antibody (Santa Cruz) for 1 h at room temperature. Protein bands were detected by enhanced chemiluminescence (ECL) Fuazon Fx (Vilber Lourmat).

### Statistical Analysis

The Student *t* test was performed to analyze the significant differences between two groups. *P* < 0.05 was considered the statistically significant.

## RESULTS

### Dox Induces Inflammation of Vascular Tissues In Vivo

**Fig. 1:**
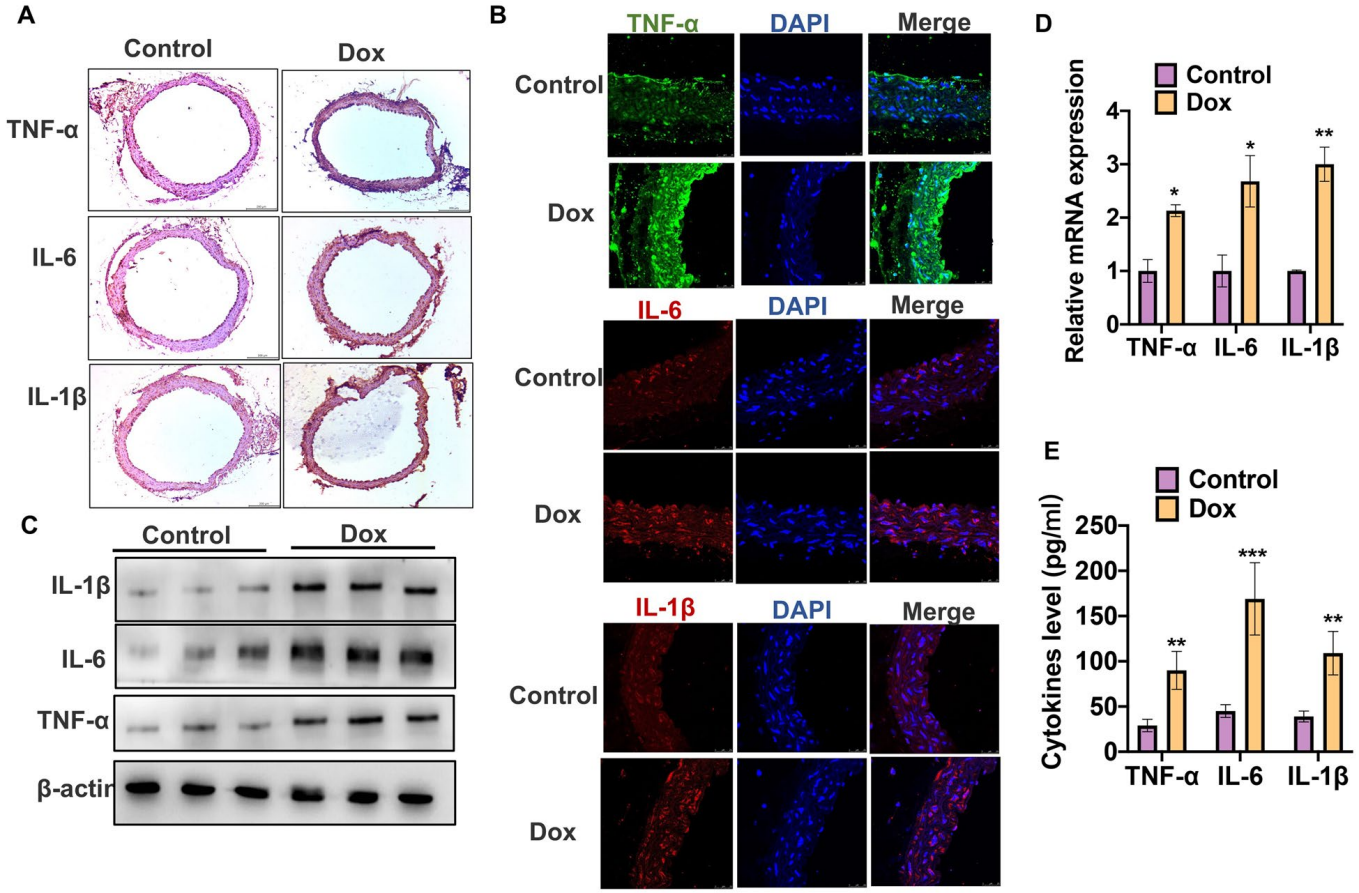
Dox induces inflammation of vascular tissues in vivo. **A** Immunohistochemical staining was used to measure the protein level of IL-1β, IL-6, and TNF-α in mice vascular tissues of Dox-treated group and control group. **B** Immunofluorescence staining was used to measure the protein level IL-1β (red), IL-6 (red), and TNF-α (green) in mice vascular tissues of Dox-treated group and control group. **C** Western blot was used to detect the protein levels of IL-1β, IL-6, and TNF-α in mice vascular tissues of Dox-treated group and control group. **D** qRT-PCR was used to detect the mRNA levels of IL-1β, IL-6, and TNF-α in Dox-treated group and control group. Normalized to β-actin. *P < 0.05, **P < 0.01 vs. control group. **E** ELISA assay was used to detect the cytokines levels of IL-1β, IL-6, and TNF-α in the mice serum. **P < 0.01, ***P < 0.001 vs. control group.

To determine the side effect of Dox in vascular tissues, we first established the Dox-induced inflammation model in vivo and then detected the key inflammatory cytokines, interleukin-6 (IL-6), interleukin-1β (IL-1β), and tumor necrosis factor-alpha (TNF-α) level in vascular tissues and mice serum. As shown in Fig. 1A, B, the immunohistochemical staining and immunofluorescence staining on mice vascular tissues showed that the expression levels of IL-1β, IL-6, and TNF-α were greatly increased in the Dox model group compared with the negative control group. Furthermore, we obtained the protein and mRNA from vascular tissues of mice. Western blot and qRT-PCR results also showed that the mRNA and protein levels of IL-1β, IL-6, and TNF-α were higher than those in negative control group (Fig. 1C, D). Moreover, we detected the level of cytokines in the serum of mice. As shown in Fig. 1E, the levels of IL-1β, IL-6, and TNF-α in Dox-treated group were increased over twofold in the mice serum compared with the negative control group. Together, these results suggest that Dox induces vascular inflammation in vivo*.*

### Dox Induces Inflammation of VSMCs In Vitro

**Fig. 2:**
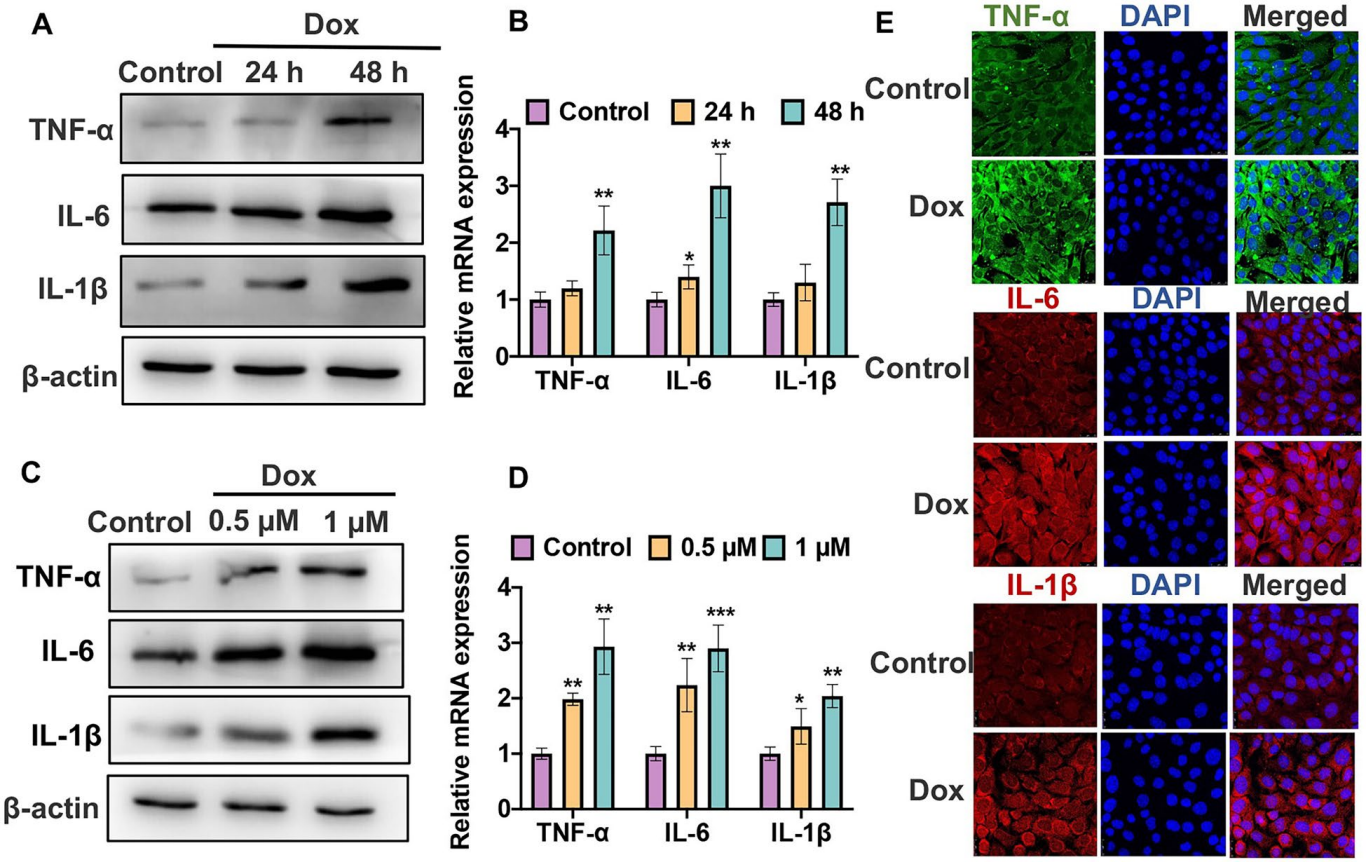
Dox induces inflammation of VSMCs in vitro. **A** VSMCs were treated with 0.5 µM Dox for 24 or 48 h. Western blot was used to detect the protein level of IL-1β, IL-6, and TNF-α. **B** Cells were treated as **A**; qRT-PCR was used to detect the protein levels of IL-1β, IL-6, and TNF-α. *P < 0.05, **P < 0.01 vs. negative control. **C** VSMCs were treated with the 0.5 or 1 µM Dox for 48 h; western blot was used to detect the protein levels of IL-1β, IL-6, and TNF-α. **D** Cells were treated as **C**; qRT-PCR was used to detect the mRNA levels of IL-1β, IL-6, and TNF-α. *P < 0.05, **P < 0.01, ***P < 0.001 vs. negative control. **E** VSMCs were treated with 1 µM Dox for 48 h. Immunofluorescence staining was used to measure the protein levels IL-1β (red), IL-6 (red), and TNF-α (green).

To further explore the effect of Dox-induced inflammation response on the VSMCs, we detected the IL-1β, IL-6, and TNFα expression in Dox-stimulated VSMCs. First, we stimulated VSMCs with 0.5 µM Dox for 24 h or 48 h. qRT-PCR and western blot results showed that stimulation of Dox markedly increased the mRNA and protein levels of IL-1β, IL-6, and TNF-α in VSMCs in a time-dependent manner compared with the negative control (Fig. 2A, B). There was a significant increase (approximately 2–threefold higher) in the levels of IL-1β, IL-6, and TNF-α in the Dox-treated-48 h group compared to the controls. Simultaneously, stimulation of Dox with 0.5 or 1 µM for 48 h led to more than a twofold increase level of IL-1β, IL-6, and TNF-α compared with the negative control. Moreover, immunofluorescence staining showed that the fluorescence intensity of TNF-α (green), IL-1β (red), and IL-6 (red) was obviously enhanced following stimulation of Dox-with 1 µM for 48 h in VSMCs (Fig. 2E). These data demonstrate that Dox could induce inflammation in VSMCs in vitro in a time- and dose-dependent manner.

### Dox Induces Inflammation in VSMCs by Moderating the PTEN/AKT/NF-κB Pathway

**Fig. 3:**
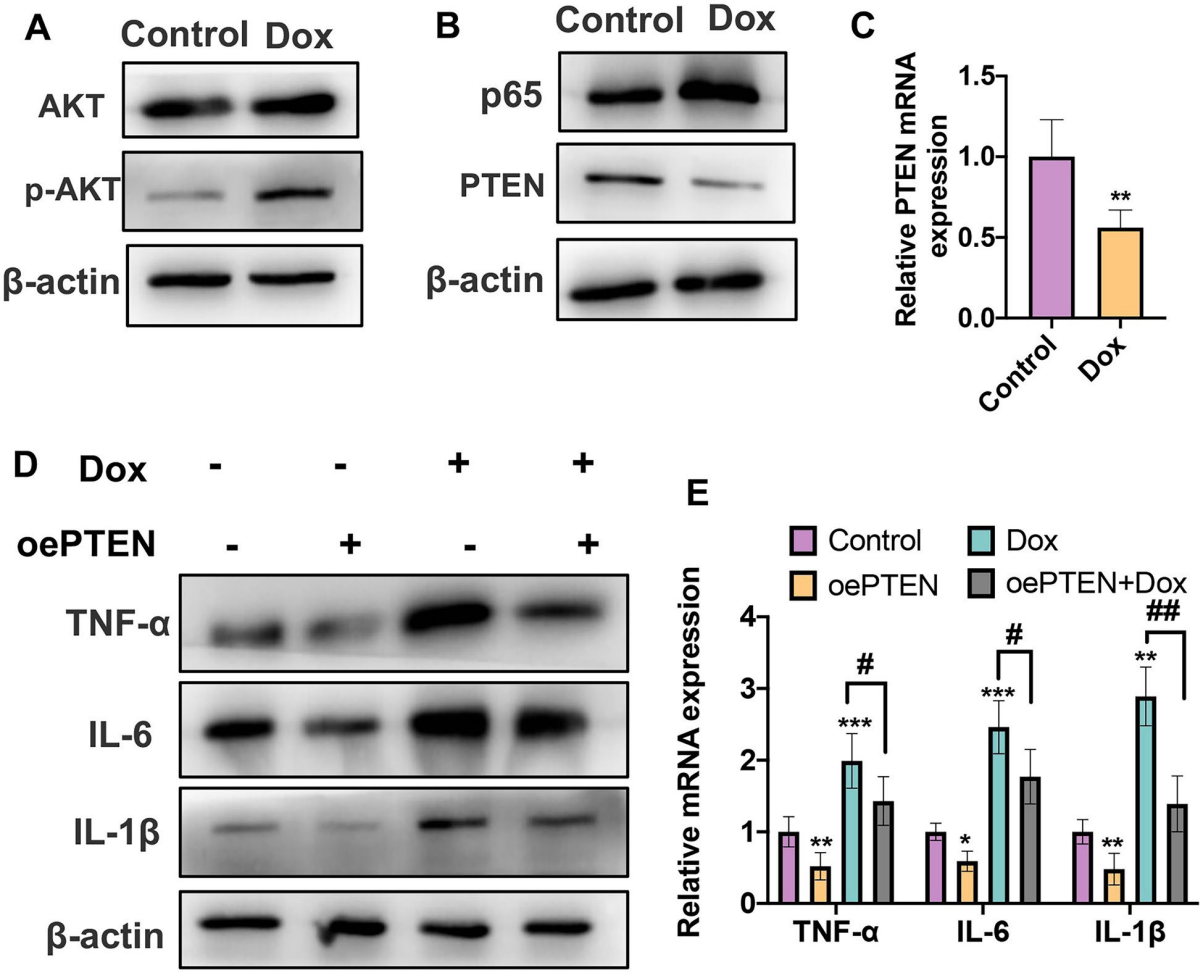
Dox induces inflammation in VSMCs by moderating the PTEN/AKT/NF-κB pathway. **A** VSMCs were treated with 1 µM Dox for 48 h. Western blot was used to detect the protein levels of AKT and p-AKT. **B** Cells were treated as **A**; western blot was used to detect the protein levels of p65 and PTEN. **C** Cells were treated as **A**; qRT-PCR was used to detect the mRNA level of PTEN. **P < 0.01 vs. negative control. **D** VSMCs were transfected with overexpression of PTEN plasmid or empty vector respectively and then treated with Dox or not. Western blot was used to detect the protein levels IL-1β, IL-6, and TNF-α. **E** Cells were treated as **D**. qRT-PCR was used to detect the protein levels of IL-1β, IL-6, and TNF-α. *P < 0.05, **P < 0.01, ***P < 0.001, # *P* < 0.05, ##*P* < 0.01 vs. corresponding control.

Previous studies have confirmed that the PTEN/AKT/NF-KB pathway plays an essential role in inflammation. Consequently, we then sought to know whether Dox could moderate the activation of PTEN/AKT/NF-KB in vitro. As shown in Fig. 3A, stimulation of Dox in VSMCs dramatically increased phosphorylated AKT (p-AKT) protein level compared with the control group, indicating that Dox could promote phosphorylation of AKT. Meanwhile, qRT-PCR and western blot analysis results showed that the stimulation of Dox to VSMCs upregulated the protein level of p65 while downregulated the mRNA and protein levels of PTEN (Fig. 3B, C). Subsequently, to further identify the role of PTEN in Dox-induced inflammation in VSMCs, we performed rescued experiments. As shown in Fig. 3D, overexpression of PTEN in VSMCs led to a decrease of the expression levels of IL-1β, IL-6, and TNF-α. In contrast, overexpression of PTEN could partly reverse Dox-induced the increase in IL-1β, IL-6, and TNF-α in both protein and mRNA levels (Fig. 3D, E). Collectively, these findings suggest that the activation of the PTEN/AKT pathway plays an important role in Dox-induced inflammation in VSMCs.

### Morin Attenuates Dox-Induced Inflammation in VSMCs

**Fig. 4:**
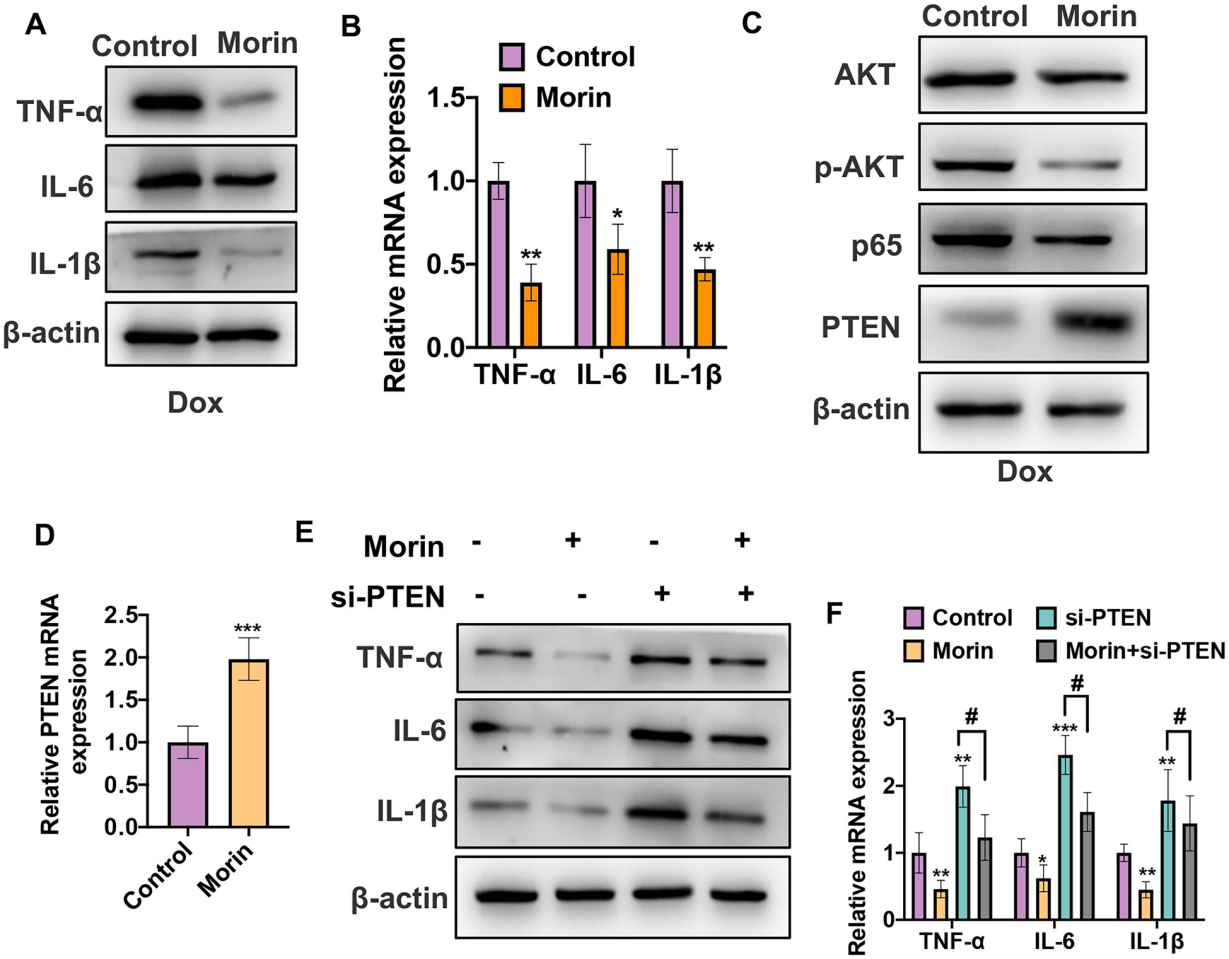
Morin attenuates Dox-induced inflammation in VSMCs. **A** VSMCs were treated with 1 µM Dox alone or combination with 25 µM Morin for 48 h. Western blot was used to detect the protein levels of IL-1β, IL-6, and TNF-α. **B** Cells were treated as **A**. qRT-PCR was used to detect the mRNA levels of IL-1β, IL-6, and TNF-α. *P < 0.05, **P < 0.01 vs. Dox alone group. **C** Cells were treated as **A**; western blot was used to detect the protein levels of AKT, p-AKT, p65, and PTEN. **D** Cells were treated as **A**; qRT-PCR was used to detect the mRNA level of PTEN. ***P < 0.001 vs. Dox alone group. **E** VSMCs were transfected with si-PTEN or si-control respectively and then treated with Morin or not. Western blot was used to detect the protein levels of IL-1β, IL-6, and TNF-α. **F** Cells were treated as **E**. qRT-PCR was used to detect the protein levels of IL-1β, IL-6, and TNF-α. *P < 0.05, **P < 0.01, ***P < 0.001, # *P* < 0.05 vs. corresponding control.

Morin (3,5,7,20,40-pentahydroxyflavone, C_15_H_10_O_7_) has the structure of one oxygen-containing heterocyclic ring connecting with two aromatic rings. Previous data showed that Morin could function as a potent anti-inflammatory agent by regulating several cell signaling pathways, especially the PTEN/AKT pathway. Therefore, we sought to investigate whether Morin could exert the anti-inflammatory effect on Dox-induced inflammation of VSMCs. To do this, first we treated VSMCs with 1 µM Dox combination with 25 µM Morin at the same time for 48 h. Western blot and qRT-PCR results showed that Morin inhibited the levels of IL-1β, IL-6, and TNF-α which were largely upregulated by Dox stimulation alone (Fig. 4A, B). Specifically, Morin could decrease the mRNA levels of TNF-α by 61%, IL-1β by 41%, and IL-6 by 53% in Dox-treated VSMCs. In addition, we detected the levels of p-AKT, p65, and PTEN by western blot and qRT-PCR. As shown in Fig. 4C, D, the protein levels of p-AKT and p65 were obviously downregulated in Morin combination treatment group compared with Dox stimulation alone. Correspondingly, PTEN expression both at the mRNA and protein levels of VSMCs was increased after treatment with Morin, indicating that Morin could suppress the activation of AKT pathway by increasing PTEN expression. To further investigate the role of PTEN in Morin-reduced VSMC inflammation, we knocked down PTEN expression in VSMCs using si-RNA and then treated with Morin or not. Western blot and qRT-PCR results showed that knockdown of PTEN induced the protein and mRNA levels of IL-1β, IL-6, and TNF-α consistent with the Dox effect, while Morin could partly reverse the induced effect by PTEN depletion in VSMCs (Fig. 4E, F). Based on the above data, Morin could decrease Dox-induced inflammation by regulating the PTEN/AKT/NF-κB pathway in VSMCs.

### miR-188-5p Mediates the Morin-Reduced Inflammation in VSMCs

**Fig. 5:**
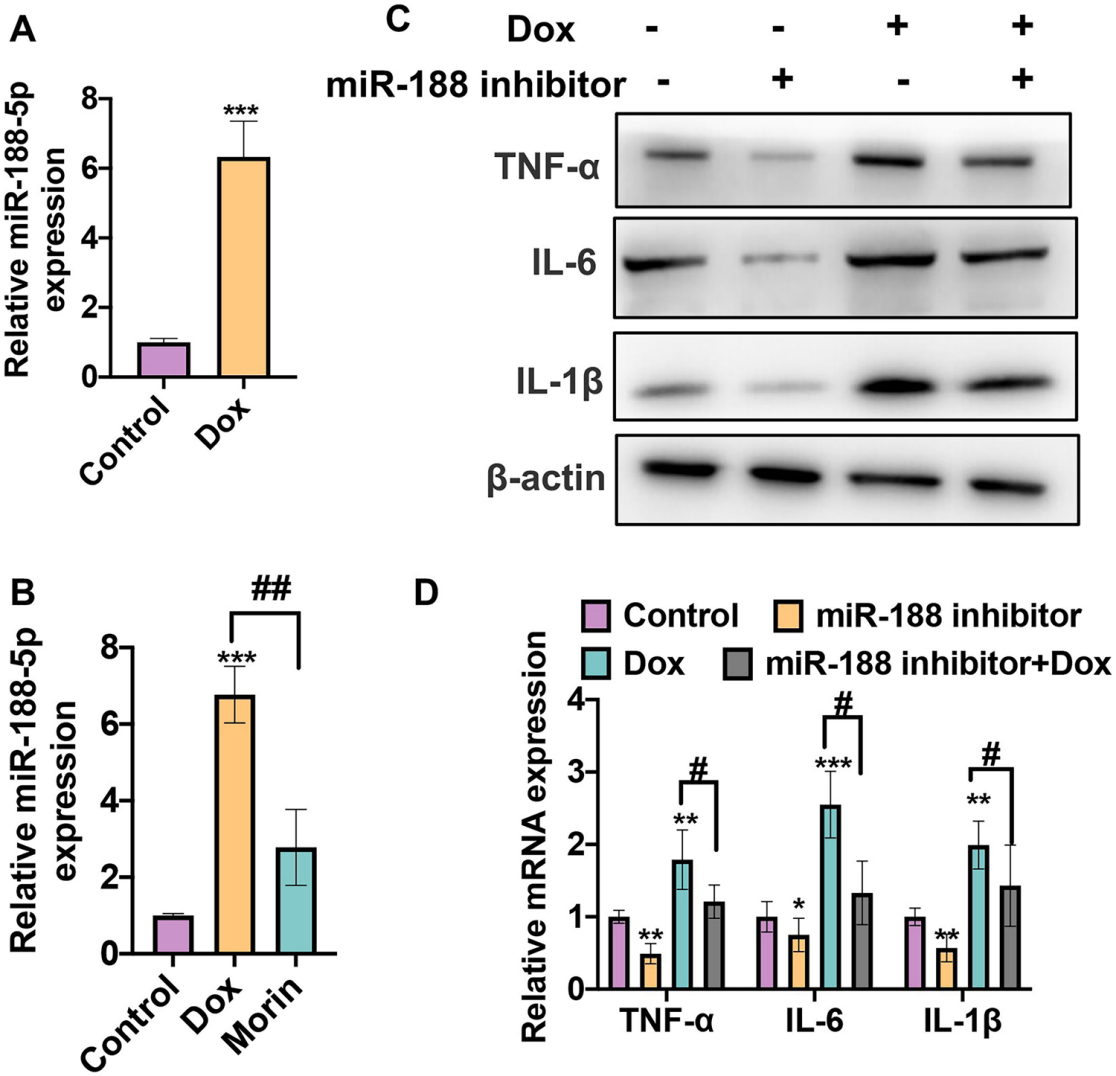
miR-188-5p mediates the Morin-reduced inflammation in VSMCs. **A** VSMCs were treated with 1 µM Dox 48 h. qRT-PCR was used to detect miR-188-5p expression. Normalized to U6. ***P < 0.001 vs. negative control.** B** VSMCs were treated with 1 µM Dox alone or combination with 25 µM Morin for 48 h. qRT-PCR was used to detect miR-188-5p expression. ***P < 0.001, # *P* < 0.05 vs. corresponding control. **C** VSMCs were transfected with miR-188-5p inhibitor or inhibitor control respectively and then treated with Dox or not. Western blot was used to detect the protein levels of IL-1β, IL-6, and TNF-α. **D** Cells were treated as **C**; qRT-PCR was used to detect the mRNA levels of IL-1β, IL-6, and TNF-α. *P < 0.05, **P < 0.01, ***P < 0.001, # *P* < 0.05 vs. corresponding control.

Our previous study has reported that miR-188-5p reduced PTEN expression by directly targeting its 3′-UTR. Morin was implicated in the regulation of the proliferation and apoptosis of chronic myelogenous leukemia (CML) cells by moderating the miR-188-5p/PTEN axis. Therefore, we hypothesized that Morin could enhance the level of PTEN by suppressing the miR-188-5p expression in Dox-induced inflammation of VSMCs. To identify this hypothesis, we stimulated the VSMCs with Dox and performed the qRT-PCR to detect the miR-188-5p expression. As Fig. 5A shows, miR-188-5p level was obviously increased sixfold in the Dox stimulation group compared with the control group. Moreover, VSMCs treated with Morin and Dox combination downregulated miR-188-5p expression which was induced by Dox alone (Fig. 5B). Then, we performed rescued experiments to test the role of miR-188-5p in Dox-induced inflammation of VSMCs. As shown in Fig. 5C, D, knockdown of miR-188-5p in VSMCs treated with Dox could partly alleviate the increase levels of cytokines caused by Dox treated alone (Fig. 5C, D). Altogether, these results indicate that miR-188-5p participates in the Dox-induced inflammation in VSMCs.

### Morin Suppresses the Dox-Induced Vascular Inflammation In Vivo

**Fig. 6:**
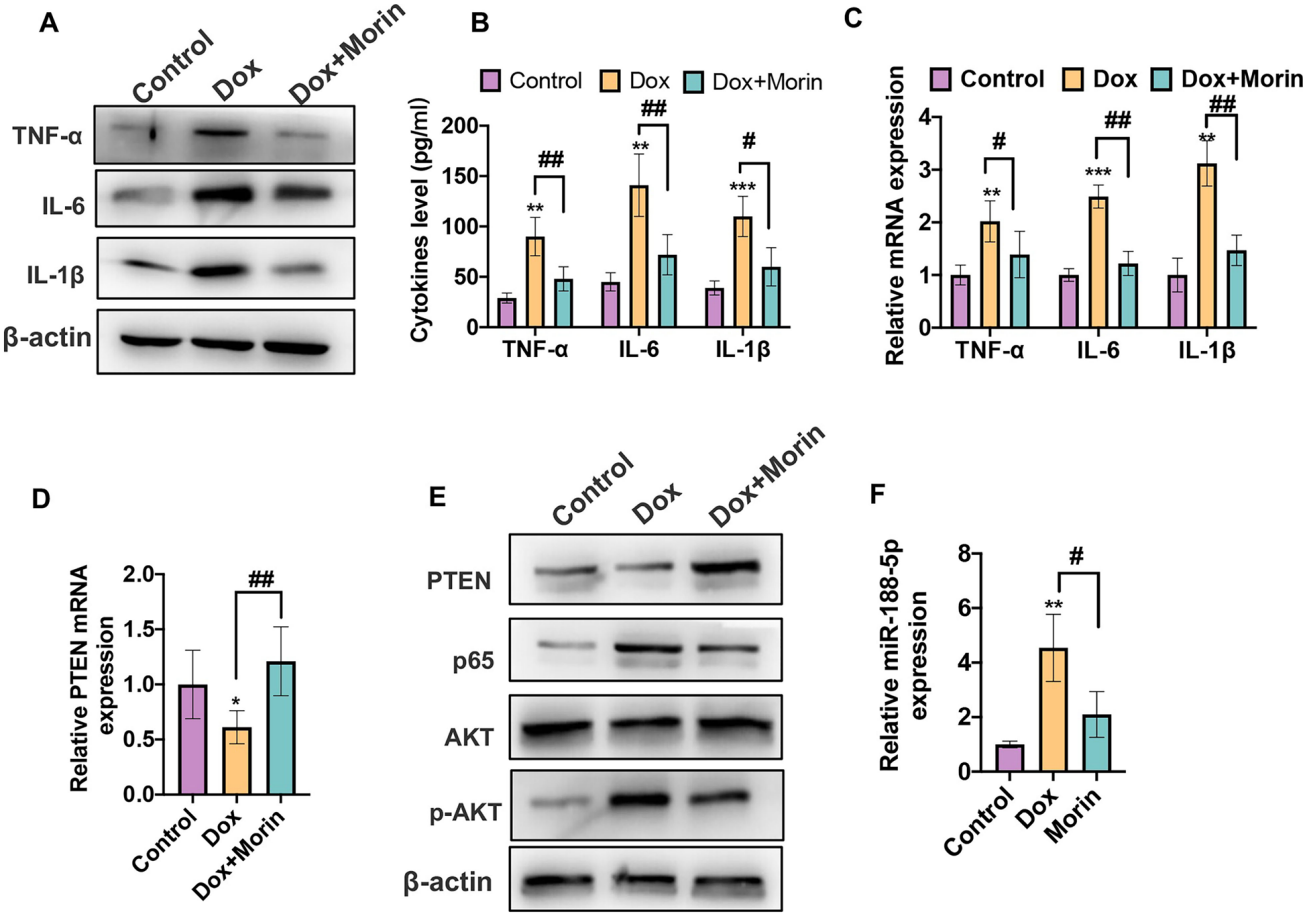
Morin suppresses the Dox-induced vascular inflammation in vivo. **A** Western blot was used to detect the protein levels of IL-1β, IL-6, and TNF-α in mice vascular tissues of Dox-treated group, Dox combination with Morin group, and control group. **B** ELISA assay was used to detect the cytokines levels of IL-1β, IL-6, and TNF-α in the mice serum of Dox-treated group, Dox combination with Morin group, and control group. **P < 0.01, ***P < 0.001, #*P* < 0.05, #*#P* < 0.01 vs. corresponding control. **C** qRT-PCR was used to detect the mRNA levels of IL-1β, IL-6, and TNF-α in mice vascular tissues of Dox-treated group, Dox combination with Morin group, and control group. **P < 0.01, ***P < 0.001, #*P* < 0.05, #*#P* < 0.01 vs. corresponding control. **D** qRT-PCR was used to detect the mRNA levels of IL-1β, IL-6, and TNF-α in mice vascular tissues. *P < 0.05, #*#P* < 0.01 vs. corresponding control. **E** Western blot was used to detect the protein levels of IL-1β, IL-6, and TNF-α in mice vascular tissues. **F** qRT-PCR was used to detect miR-188-5p expression in mice vascular tissues. **P < 0.01, *#P* < 0.05 vs. corresponding control.

To further confirm the above findings that Morin could suppress the Dox-induced inflammation in VSMCs, we performed in vivo experiments as follows. Mice were either treated with Dox respectively or co-treated with Dox and Morin together. First, we assessed the expression of inflammatory cytokine in vascular tissues. As shown in Fig. 6A, B, Morin effectively decreased the levels of IL-1β, IL-6, and TNF-α by more than 50% in both protein and mRNA levels, which were increased by Dox treatment. Consistent with the above results, ELISA analysis showed that treatment with Morin could reduce the level of cytokines in serum compared with the Dox alone group (Fig. 6C). Subsequently, we detected whether the activation of the PTEN/AKT pathway was changed in the vascular tissues of Dox-induced inflammation model. As shown in Fig. 6E, the mRNA and protein levels of PTEN were both downregulated in the Dox alone group, while this inhibition effect was reversed in the co-treatment with Morin group. Furthermore, treatment with Morin decreased the expression of p-AKT and p65 in the vascular tissues compared with the Dox group (Fig. 6D). Importantly, qRT-PCR result showed that treatment with Dox induced a fourfold or greater upregulation of the miR-188-5p level, whereas the increased effect could be downregulated when co-treating with Morin (Fig. 6F). The above data strongly suggest that Morin could inhibit Dox-induced inflammation by regulating the miR-188-5p/PTEN/AKT/NF-κB pathway in VSMCs.

## DISCUSSION

In the present study, we revealed several key findings as follows: (1) we identified that Dox could promote the inflammation in vascular tissues of the Dox-treated mouse model and in VSMCs. (2) Dox significantly downregulated the PTEN expression level and increased the AKT phosphorylation, thus regulating P65 level. (3) Morin, as an indirect activator of PTEN, increased PTEN level which in turn inhibited AKT pathway activation. (4) Morin plays an anti-inflammatory role in Dox-induced vascular inflammation by regulating miR-188-5p/PTEN/AKT pathway.

Previous studies have confirmed that Morin is a potentially anti-infective agent that has a significant anti-inflammatory effect in many infectious diseases. For example, treatment with Morin in LPS-induced mastitis and LPS-induced acute lung injury significant decreased the expression of inflammatory cytokines, including TNF-α, IL-1β, and IL-6. Moreover, the authors found that Morin could inhibit the activation of the NF-κB pathway in LPS-induced mastitis [24, 25]. Additionally, Morin could reduce the TNF-α and IL-6 levels by inhibiting the PI3K/AKT/NF-κB pathway atherosclerosis which considered a chronic inflammatory disease [26]. These findings were consistent with our results.

PI3K/AKT/NF-κB pathway is a crucial and quite complex pathway which is involved in physiological functions, including cell survival, proliferation, metabolism, apoptosis, and differentiation [27–29]. PI3K/AKT activation serves an essential role in inflammatory responses [30, 31]. Additionally, activation of the PI3K/AKT pathway is closely related with the NF-κB pathway. On the one hand, AKT phosphorylation could promote NF-κB activation by affecting IκB kinase activity as well as phosphorylation and nuclear translocation of p65. On the other hand, as an important transcription factor, activation of NF-κB could transcriptionally regulate the protein level of PI3K/AKT pathway, and thus contribute to its subsequent activity [32, 33]. In the present study, we revealed that Dox could accentuate the cytokine levels in serum. We speculated that the increase in proinflammatory factors was secreted by macrophages or other inflammatory cells*.* These proinflammatory factors, such as IL-1β, could activate AKT phosphorylation and NF-κB activation of VSMCs. Stimulated VSMCs secreted more cytokines, rendering the inflammation expanded and making a feedback loop. Our data validated that IL-1β, IL-6, and TNF-α were increased in VSMCs and vascular tissues after treated with the Dox in vitro and in vivo, respectively. Moreover, we found that the phosphorylation of AKT and p65 were upregulated by Dox treatment mean that the activation of AKT/NF-κB pathway. More importantly, we also demonstrated that the PTEN i was inhibited by Dox treatment and thus could help to account for the activation of AKT/NF-κB pathway. Despite the fact that our findings elucidated the mechanism of Dox-induced inflammation of in vascular tissues, this study has several limitations. One of the limitations is that we did not investigate the macrophage behavior in the Dox-treated animal model and cells. However, this might form the basis for future research*.*

PTEN is a crucial negative regulator of the PI3K/AKT pathway that can dephosphorylate phosphatidylinositol 3,4,5-triphosphate (PIP_3_) to phosphatidylinositol 4,5-biphosphate (PIP_2_). As an important tumor suppressor, depletion of PTEN promotes susceptibility to tumorigenesis and contributes to tumor cell proliferation, apoptosis, and cell survival and metabolism [34, 35]. Apart from this, PTEN is confirmed to be involved in the inflammatory response. For example, in the IL-10^−/−^ mouse model, overexpression of PTEN suppressed the flagellin-promoted colonic inflammation in epithelial cells by disrupting Mal-TLR5 interaction [36]. In toluene diisocyanate-induced asthma model, upregulation of PTEN could reduce allergen-induced airway inflammation by inhibiting of IL-17 expression [37]. In ischemia–reperfusion injury mouse model, depletion of PTEN by its inhibitor significantly expanded the inflammation and promoted acute kidney injury [38]. In the LPS-induced ameliorates lung damage mouse model, knockdown of PTEN attenuated LPS-induced lung inflammation by regulating the β-catenin pathway [39]. In our present study, we demonstrated that PTEN played a crucial role in Dox-induced vascular inflammation mouse model and Dox-treated VSMCs. Dox treatment decreased PTEN expression in vivo and in vitro, and PTEN upregulation could reduce the release of inflammatory cytokines which was induced by Dox. Our findings indicate that PTEN activation might be a potential therapeutic option in Dox-induced vascular inflammation.

The proven mechanisms in loss-function of PTEN include mutation statue, transcription regulation, posttranscriptional regulation, and epigenetic regulation*.* For example, the high expression of DNA methyltransferase 1 (DNMT1) in breast cancer cell influenced of methylation in the PTEN promoter thus leading to the loss of PTEN [40]*.* Furthermore, the SALL4-NuRD complex in 293 T cells enhanced the histone hyperacetylation in PTEN promotor, resulting in the depletion of PTEN expression [41]. Dysregulation of NEDD4 in glioblastoma cells regulated PTEN expression by promoting its ubiquitination and degradation [42]. Moreover, associated studies have confirmed that the transcription factors, such as p53, peroxisome proliferator activated receptor gamma (PPARγ), and early growth response 1 (EGR1), positively regulated PTEN expression by binding to its promotor [43–45]. Our previous study observed that PTEN was negatively regulated by miR-188-5p which was overexpressed in CML cells [22]. Moreover, Morin was found to inhibit miR-188-5p expression in CML cells, resulting in the upregulation of PTEN expression. Consistent with the previous research, upregulation of miR-188-5p was observed in the Dox-treated VSMCs and mouse model. Knockdown of miR-188-5p in Dox-treated VSMCs could reduce the inflammation which had the same effect as PTEN overexpression. Of importance, we further confirmed that miR-188-5p was decreased whereas PTEN was increased after Morin treatment in vivo and in vitro. Therefore, we hypothesized that Morin might be participating in the effects of Dox-induced vascular inflammation and anti-leukemia by the common molecular mechanism. Alternatively, our results also suggested that the dysregulation of the miR-188-5p/PTEN pathway might exert an important role in Dox-induced inflammation as well as CML tumorigenesis. However, whether other mechanisms were involved in Morin-reduced inflammation needs further investigation.

## CONCLUSION

In conclusion, as a PTEN indirectly activator, Morin reduced the Dox-induced vascular inflammation by moderating the miR-188-5p/PTEN/AKT/NF-κB pathway. Hence, Morin may have a therapeutic value in overcoming the chemotherapy side effects in the future.

## Data Availability

The data used or analyzed during the current study are available from the corresponding author on reasonable request.

## Abbreviations

Dox: Doxorubicin
IL-6: Interleukin-6
IL-1β: Interleukin-1β
TNF-α: Tumor necrosis factor-alpha
VSMCs: Vascular smooth muscle cells
PTEN: Phosphatase and tension homology deleted on chromosome 10
NF-κB: Nuclear factor kappa B
AKT: Protein kinase B
PI3K: Phosphatidylinositol-3-kinase
CML: Chronic myelogenous leukemia
ELISA: Enzyme-linked immunosorbent assay
RT-qPCR: Reverse transcription-quantitative polymerase chain reaction

## References

[1] Siegel RL, Miller KD, Jemal A (2020). Cancer statistics, 2020. CA: A Cancer Journal for Clinicians.

[2] Yang X, Wang J (2018). Precision therapy for acute myeloid leukemia. Journal of Hematology & Oncology.

[3] Yang S, Zhang Z, Wang Q (2019). Emerging therapies for small cell lung cancer. Journal of Hematology & Oncology.

[4] Pirker R (2020). Chemotherapy remains a cornerstone in the treatment of nonsmall cell lung cancer. Current Opinion in Oncology.

[5] Heinhuis KM, Ros W, Kok M, Steeghs N, Beijnen JH, Schellens JHM (2019). Enhancing antitumor response by combining immune checkpoint inhibitors with chemotherapy in solid tumors. Annals of Oncology.

[6] Abu Zaid M, Dinh PC, Monahan PO, Fung C, El-Charif O, Feldman DR, Hamilton RJ, Vaughn DJ, Beard CJ, Cook R (2019). Adverse health outcomes in relationship to hypogonadism after chemotherapy: A multicenter study of testicular cancer survivors. Journal of the National Comprehensive Cancer Network.

[7] Adams SC, DeLorey DS, Davenport MH, Fairey AS, North S, Courneya KS (2018). Effects of high-intensity interval training on fatigue and quality of life in testicular cancer survivors. British Journal of Cancer.

[8] Zraik IM, Heß-Busch Y (2021). Management of chemotherapy side effects and their long-term sequelae. Urologe A.

[9] Rivankar S (2014). An overview of doxorubicin formulations in cancer therapy. Journal of Cancer Research and Therapeutics.

[10] Wenningmann N, Knapp M, Ande A, Vaidya TR, Ait-Oudhia S (2019). Insights into doxorubicin-induced cardiotoxicity: Molecular mechanisms, preventive strategies, and early monitoring. Molecular Pharmacology.

[11] Brewster DH, Clark D, Hopkins L, Bauer J, Wild SH, Edgar AB, Wallace WH (2014). Subsequent hospitalisation experience of 5-year survivors of childhood, adolescent, and young adult cancer in Scotland: A population based, retrospective cohort study. British Journal of Cancer.

[12] Lv H, Tan R, Liao J, Hao Z, Yang X, Liu Y, Xia Y (2020). Doxorubicin contributes to thrombus formation and vascular injury by interfering with platelet function. American Journal of Physiology. Heart and Circulatory Physiology.

[13] Pugazhendhi A, Edison T, Velmurugan BK, Jacob JA, Karuppusamy I (2018). Toxicity of doxorubicin (Dox) to different experimental organ systems. Life Sciences.

[14] Stojanović SD, Fiedler J, Bauersachs J, Thum T, Sedding DG (2020). Senescence-induced inflammation: An important player and key therapeutic target in atherosclerosis. European Heart Journal.

[15] Stojanović SD, Fuchs M, Kunz M, Xiao K, Just A, Pich A, Bauersachs J, Fiedler J, Sedding D, Thum T (2020). Inflammatory drivers of cardiovascular disease: Molecular characterization of senescent coronary vascular smooth muscle cells. Frontiers in Physiology.

[16] Qi D, Wei M, Jiao S, Song Y, Wang X, Xie G, Taranto J, Liu Y, Duan Y, Yu B (2019). Hypoxia inducible factor 1α in vascular smooth muscle cells promotes angiotensin II-induced vascular remodeling via activation of CCL7-mediated macrophage recruitment. Cell Death & Disease.

[17] Jin QS, Huang LJ, Zhao TT, Yao XY, Lin LY, Teng YQ, Kim SH, Nam MS, Zhang LY, Jin YJ (2018). HOXA11-AS regulates diabetic arteriosclerosis-related inflammation via PI3K/AKT pathway. European Review for Medical and Pharmacological Sciences.

[18] Caselli A, Cirri P, Santi A, Paoli P (2016). Morin: A promising natural drug. Current Medicinal Chemistry.

[19] Sinha K, Ghosh J, Sil PC (2016). Morin and its role in chronic diseases. Advances in Experimental Medicine and Biology.

[20] Jiang A, Zhang Y, Zhang X, Wu D, Liu Z, Li S, Liu X, Han Z, Wang C, Wang J (2020). Morin alleviates LPS-induced mastitis by inhibiting the PI3K/AKT, MAPK, NF-κB and NLRP3 signaling pathway and protecting the integrity of blood-milk barrier. International Immunopharmacology.

[21] Rajput SA, Wang XQ, Yan HC (2021). Morin hydrate: A comprehensive review on novel natural dietary bioactive compound with versatile biological and pharmacological potential. Biomedicine & Pharmacotherapy.

[22] Nie ZY, Yang L, Liu XJ, Yang Z, Yang GS, Zhou J, Qin Y, Yu J, Jiang LL, Wen JK (2019). Morin inhibits proliferation and induces apoptosis by modulating the miR-188-5p/PTEN/AKT regulatory pathway in CML cells. Molecular Cancer Therapeutics.

[23] Zheng B, Zheng CY, Zhang Y, Yin WN, Li YH, Liu C, Zhang XH, Nie CJ, Zhang H, Jiang W (2018). Regulatory crosstalk between KLF5, miR-29a and Fbw7/CDC4 cooperatively promotes atherosclerotic development. Biochimica et Biophysica Acta, Molecular Basis of Disease.

[24] Yu S, Liu X, Yu D, Changyong E, Yang J (2020). Morin protects LPS-induced mastitis via inhibiting NLRP3 inflammasome and NF-κB signaling pathways. Inflammation.

[25] Tianzhu Z, Shihai Y, Juan D (2014). The effects of Morin on lipopolysaccharide-induced acute lung injury by suppressing the lung NLRP3 inflammasome. Inflammation.

[26] Meng Q, Pu L, Lu Q, Wang B, Li S, Liu B, Li F (2021). Morin hydrate inhibits atherosclerosis and LPS-induced endothelial cells inflammatory responses by modulating the NFκB signaling-mediated autophagy. International Immunopharmacology.

[27] DiDonato JA, Mercurio F, Karin M (2012). NF-κB and the link between inflammation and cancer. Immunological Reviews.

[28] Wang J, Fu D, Senouthai S, You Y (2019). Critical roles of PI3K/Akt/NF-κB survival axis in angiotensin II-induced podocyte injury. Molecular Medicine Reports.

[29] Xu F, Na L, Li Y, Chen L (2020). Roles of the PI3K/AKT/mTOR signalling pathways in neurodegenerative diseases and tumours. Cell & Bioscience.

[30] Vergadi E, Ieronymaki E, Lyroni K, Vaporidi K, Tsatsanis C (2017). Akt signaling pathway in macrophage activation and M1/M2 polarization. The Journal of Immunology.

[31] Hawkins PT, Stephens LR (2015). PI3K signalling in inflammation. Biochimica et Biophysica Acta.

[32] Qu R, Chen X, Wang W, Qiu C, Ban M, Guo L, Vasilev K, Chen J, Li W, Zhao Y (2018). Ghrelin protects against osteoarthritis through interplay with Akt and NF-κB signaling pathways. The FASEB Journal.

[33] Balwani S, Chaudhuri R, Nandi D, Jaisankar P, Agrawal A, Ghosh B (2012). Regulation of NF-κB activation through a novel PI-3K-independent and PKA/Akt-dependent pathway in human umbilical vein endothelial cells. PLoS ONE.

[34] Downes CP, Ross S, Maccario H, Perera N, Davidson L, Leslie NR (2007). Stimulation of PI 3-kinase signaling via inhibition of the tumor suppressor phosphatase. PTEN. Adv Enzyme Regul.

[35] Chen CY, Chen J, He L, Stiles BL (2018). PTEN: Tumor suppressor and metabolic regulator. Front Endocrinol (Lausanne).

[36] Choi YJ, Jung J, Chung HK, Im E, Rhee SH (2013). PTEN regulates TLR5-induced intestinal inflammation by controlling Mal/TIRAP recruitment. The FASEB Journal.

[37] Kim SR, Lee KS, Park SJ, Min KH, Lee KY, Choe YH, Lee YR, Kim JS, Hong SJ, Lee YC (2007). PTEN down-regulates IL-17 expression in a murine model of toluene diisocyanate-induced airway disease. The Journal of Immunology.

[38] Zhou J, Jia L, Hu Z, Wang Y (2017). Pharmacological inhibition of PTEN aggravates acute kidney injury. Science and Reports.

[39] Zhou M, Fang H, Du M, Li C, Tang R, Liu H, Gao Z, Ji Z, Ke B, Chen XL (2019). The modulation of regulatory T cells via HMGB1/PTEN/β-catenin axis in LPS induced acute lung injury. Frontiers in Immunology.

[40] Giannoudis A, Malki MI, Rudraraju B, Mohhamed H, Menon S, Liloglou T, Ali S, Carroll JS, Palmieri C (2020). Activating transcription factor-2 (ATF2) is a key determinant of resistance to endocrine treatment in an in vitro model of breast cancer. Breast Cancer Research.

[41] Lu J, Jeong HW, Kong N, Yang Y, Carroll J, Luo HR, Silberstein LE (2009). Yupoma, Chai L: **Stem cell factor SALL4 represses the transcriptions of PTEN and SALL1 through an epigenetic repressor complex**. PLoS ONE.

[42] Xia Q, Ali S, Liu L, Li Y, Liu X, Zhang L, Dong L (2020). Role of ubiquitination in PTEN cellular homeostasis and its implications in GB drug resistance. Frontiers in Oncology.

[43] Virolle T, Adamson ED, Baron V, Birle D, Mercola D, Mustelin T, de Belle I (2001). The Egr-1 transcription factor directly activates PTEN during irradiation-induced signalling. Nature Cell Biology.

[44] Stambolic V, MacPherson D, Sas D, Lin Y, Snow B, Jang Y, Benchimol S, Mak TW (2001). Regulation of PTEN transcription by p53. Molecular Cell.

[45] Patel L, Pass I, Coxon P, Downes CP, Smith SA, Macphee CH (2001). Tumor suppressor and anti-inflammatory actions of PPARgamma agonists are mediated via upregulation of PTEN. Current Biology.

